# Clinical laboratory tests and five-year incidence of major depressive disorder: a prospective cohort study of 433,890 participants from the UK Biobank

**DOI:** 10.1038/s41398-021-01505-5

**Published:** 2021-07-07

**Authors:** Michael Wainberg, Stefan Kloiber, Breno Diniz, Roger S. McIntyre, Daniel Felsky, Shreejoy J. Tripathy

**Affiliations:** 1 Centre for Addiction and Mental Health, Toronto, ON Canada; 2 Krembil Centre for Neuroinformatics, Centre for Addiction and Mental Health, Toronto, ON Canada; 3 Institute of Medical Sciences, University of Toronto, Toronto, ON Canada; 4 Department of Psychiatry, University of Toronto, Toronto, ON Canada; 5 Mood Disorders Psychopharmacology Unit, University Health Network, Toronto, ON Canada; 6 Department of Physiology, University of Toronto, Toronto, ON Canada

**Keywords:** Biomarkers, Depression

## Abstract

Prevention of major depressive disorder (MDD) is a public health priority. Identifying biomarkers of underlying biological processes that contribute to MDD onset may help address this public health need. This prospective cohort study encompassed 383,131 white British participants from the UK Biobank with no prior history of MDD, with replication in 50,759 participants of other ancestries. Leveraging linked inpatient and primary care records, we computed adjusted odds ratios for 5-year MDD incidence among individuals with values below or above the 95% confidence interval (<2.5th or >97.5th percentile) on each of 57 laboratory measures. Sensitivity analyses were performed across multiple percentile thresholds and in comparison to established reference ranges. We found that indicators of liver dysfunction were associated with increased 5-year MDD incidence (even after correction for alcohol use and body mass index): elevated alanine aminotransferase (AOR = 1.35, 95% confidence interval [1.16, 1.58]), aspartate aminotransferase (AOR = 1.39 [1.19, 1.62]), and gamma glutamyltransferase (AOR = 1.52 [1.31, 1.76]) as well as low albumin (AOR = 1.28 [1.09, 1.50]). Similar observations were made with respect to endocrine dysregulation, specifically low insulin-like growth factor 1 (AOR = 1.34 [1.16, 1.55]), low testosterone among males (AOR = 1.60 [1.27, 2.00]), and elevated glycated hemoglobin (HbA1C; AOR = 1.23 [1.05, 1.43]). Markers of renal impairment (i.e. elevated cystatin C, phosphate, and urea) and indicators of anemia and macrocytosis (i.e. red blood cell enlargement) were also associated with MDD incidence. While some immune markers, like elevated white blood cell and neutrophil count, were associated with MDD (AOR = 1.23 [1.07, 1.42]), others, like elevated C-reactive protein, were not (AOR = 1.04 [0.89, 1.22]). The 30 significant associations validated as a group in the multi-ancestry replication cohort (Wilcoxon *p* = 0.0005), with a median AOR of 1.235. Importantly, all 30 significant associations with extreme laboratory test results were directionally consistent with an increased MDD risk. In sum, markers of liver and kidney dysfunction, growth hormone and testosterone deficiency, innate immunity, anemia, macrocytosis, and insulin resistance were associated with MDD incidence in a large community-based cohort. Our results support a contributory role of diverse biological processes to MDD onset.

## Introduction

Major depressive disorder (MDD) is associated with systemic as well as central dysfunction. The promise that better understanding this systemic dysfunction might lead to improved preventative and disease-modifying interventions has spurred interest in uncovering peripheral biomarkers of MDD [1–3] and its subtypes [4], treatment response [5], and prediction of future episodes [6]. Among the most frequently implicated are inflammatory markers like C-reactive protein (CRP) and cytokines [7], insulin resistance markers [8, 9], hormones [10–13], neurotrophic factors like brain-derived neurotrophic factor [14, 15], neurotransmitters (and their metabolites) [16], and members of the kynurenine pathway [17]. While some of these associations are likely reflective of comorbid somatic disease, considering biomarkers rather than formal physician diagnoses gets closer to the underlying biology and is less prone to diagnostic bias.

Despite efforts to find biochemical markers that predict future MDD episodes, finding ones that are robustly replicable has remained a challenge. Publication bias [4, 18], confounding [6], small sample size, and methodological heterogeneity across studies [4, 5], all complicate the interpretation of prior studies on this topic. Meta-analyses cannot fully mitigate these concerns, and recent meta-analyses have failed to identify replicable biomarkers predictive of MDD incidence. A recent meta-analysis observed only a single association between cortisol and MDD incidence that did not survive multiple testing correction [6].

To better understand which types of somatic dysfunction might contribute to MDD onset, we set out to identify robust biomarkers of incident MDD. We turned to the UK Biobank, a prospective cohort study of 502,617 British individuals that includes several dozen common laboratory tests. The UK Biobank’s comprehensive characterization of participants enables analyses with large sample size, consistent methodology across a wide variety of markers, and rigorous correction for covariates using the rich sociodemographic and clinical data available on each participant. Furthermore, its linkage to inpatient and primary care records allows for a prospective study design focused on new-onset MDD diagnoses among individuals with no prior history of MDD. We reasoned that these unique aspects might mitigate some of the limitations that have confounded existing cross-sectional MDD biomarker studies [6].

## Methods

### Participants

Participants were drawn from the UK Biobank, a community-based prospective cohort study of 502,617 British individuals, aged 40–69 at recruitment. In total, 68,727 participants were excluded due to lacking all laboratory test results or any covariates, or already having evidence of an MDD diagnosis at the time of the initial assessment (based on Data-Fields #130894, “Date F32 first reported (depressive episode)”, and #130896, “Date F33 first reported (recurrent depressive disorder)”). In total, 433,890 individuals remained in the final cohort: of these, 383,131 of self-reported white British ancestry (Data-Field #21000, “Ethnic background”) were used in the main analysis), and the remaining 50,759 of other ancestries for replication.

### Outcome definition

The outcome was an F32 ICD-10 diagnosis code from linked inpatient, primary care, or death records within 5 years of initial assessment (“Date F32 first reported (depressive episode)”, Data-Field #130894, along with “Source of report of F32 (depressive episode)”, Data-Field #130895, to exclude cases supported only by self-report). In all, 5534 participants (4851 white British, 683 of other ancestries) met this criterion and were deemed incident MDD cases, with the remaining 428,356 participants (378,280 white British, 50,076 of other ancestries) deemed controls. Among the white British cases, MDD diagnosis occurred a mean of 2.7 (standard deviation 1.4) years after laboratory testing.

### Laboratory tests

30 blood analytes and 31 blood counts (Data-Fields tab of https://biobank.ctsu.ox.ac.uk/crystal/label.cgi?id=17518 and https://biobank.ctsu.ox.ac.uk/crystal/label.cgi?id=100081) were measured at initial assessment (Fig. S1). The UK Biobank performed detailed quality control (QC) and correction for technical outliers (details for blood analytes and counts available at https://biobank.ctsu.ox.ac.uk/crystal/crystal/docs/serum_biochemistry.pdf and https://biobank.ctsu.ox.ac.uk/crystal/crystal/docs/haematology.pdf, respectively). We analyzed 57 of these 61 tests, after excluding rheumatoid factor, estradiol, and nucleated red blood cell count and percentage, where most participants had values of 0 or values outside the reportable range according to the UK Biobank’s QC. We stratified testosterone and sex-hormone-binding globulin analyses by sex.

Following the UK Biobank (https://biobank.ctsu.ox.ac.uk/~bbdatan/biomarkers.pdf), we categorized each blood biochemistry test as either “renal” (i.e. cystatin C, creatinine, phosphate, total protein, urate, urea), “liver” (i.e. alanine and aspartate aminotransferase, gamma glutamyltransferase, albumin, direct bilirubin, total bilirubin), “bone and joint” (i.e. alkaline phosphatase, calcium, vitamin D), “diabetes” (i.e. glucose, HbA1C), “cancer” (i.e. sex-hormone-binding globulin, testosterone, IGF-1), or “cardiovascular (i.e. CRP, apolipoproteins A and B, lipoprotein A, triglycerides, total cholesterol, HDL cholesterol, LDL cholesterol). However, we rename the “cancer” category to “endocrine”, since all markers in this category are endocrine markers, and combine “cardiovascular” and “diabetes” into a single “immunometabolic” category. We note that these categories are somewhat simplistic, as many markers reflect dysfunction in multiple categories: for instance, low albumin and total protein may reflect either liver or renal dysfunction, and high alkaline phosphatase may reflect either liver or skeletal dysfunction.

### Covariates

Due to the strong influence on covariates on our tests [19], several types were included in the analysis. Demographic covariates comprised age at initial assessment (Data-Field #21003), sex (#31), location of UK Biobank assessment center (#54) as a 21-level categorical variable, self-reported ethnic background (#21000) as a 6-level categorical variable (included for replication only; white, mixed, Asian or Asian British, Black or Black British, Chinese, other ethnic group), and the top 10 genotype principal components (a proxy for genetic ancestry; #22009). Temporal covariates comprised season and time of day of the blood draw (both derived from #3166) and hours fasted before the blood draw (#74). Socioeconomic covariates comprised educational qualifications (#6138) as a 6-level categorical variable, pre-tax household income bracket (#738) as a 5-level categorical variable, employment status (#6142) as a 7-level categorical variable, Townsend deprivation index (#189), and Index of Multiple Deprivation (#26410/26427/26426 for England/Scotland/Wales). Finally, lifestyle covariates comprised alcohol intake frequency (#1558) as a 5-level categorical variable, smoking status (never/current/previous; #20116), and body mass index (#21001). Selected covariate frequencies among incident MDD cases and controls are shown in Table S1.

### Statistical analysis

Each blood test was associated with incident MDD across all individuals with valid data for that test. Tests were dichotomized in two different ways: first, individuals with test values in the bottom 2.5% were compared to those in the top 97.5%; second, individuals with test values in the top 2.5% were compared to those in the bottom 97.5%. For each dichotomization, incident MDD was logistically regressed against the dichotomized blood test and covariates to yield adjusted odds ratios (AORs) and associated 95% confidence intervals, using the *statsmodels* Python package [20]. Non-binary covariates were standardized to zero mean and unit variance; to avoid convergence issues, binary covariates with <5% frequency in either cases or controls were excluded. False discovery rate correction was performed across the 118 tests conducted (57 blood tests, of which two are sex-specific, times two dichotomizations).

### Reference ranges

We obtained reference ranges from a mix of sources (Table S2). As the UK Biobank only provides reference ranges for blood counts (https://biobank.ctsu.ox.ac.uk/crystal/crystal/docs/haematology.pdf), we used the Oxford University Hospitals’ (https://www.ouh.nhs.uk/biochemistry/tests/documents/biochemistry-reference-ranges.pdf) for biochemistry tests (except Cystatin C’s, which was not listed and obtained from the literature instead [21]). For tests with age- or sex-specific reference ranges (e.g. IGF-1), we used the most extreme listed threshold.

## Results

### Association of blood biochemistry tests with 5-year MDD incidence

We leveraged the UK Biobank’s longitudinal nature to test whether white British participants (*N* = 383,131) outside the 95% confidence interval (i.e. <2.5th or >97.5th percentile among the full cohort; Table 1) on each of 57 blood tests at initial assessment were diagnosed with MDD at greater rates during the following 5 years, accounting for demographic, temporal, socioeconomic, and lifestyle covariates (see “Methods”).

**Table 1:** 2.5th and 97.5th percentile thresholds for each blood test across the full cohort.

Blood biochemistry	2.5th %ile	97.5th %ile	Blood count	2.5th %ile	97.5th %ile
Alanine aminotransferase (ALT)	9.5 U/L	57.4 U/L	Basophil count	0 × 10^9^/L	0.12 × 10^9^/L
Albumin	40.2 g/L	50.4 g/L	Basophil percentage	0.1%	1.7%
Alkaline phosphatase (ALP)	46.6 U/L	138 U/L	Eosinophil count	0 × 10^9^/L	0.5 × 10^9^/L
Apolipoprotein A (ApoA)	1.1 g/L	2.2 g/L	Eosinophil percentage	0.5%	7.2%
Apolipoprotein B (ApoB)	0.6 g/L	1.6 g/L	Hematocrit percentage	34.4%	48.0%
Aspartate aminotransferase (AST)	16.0 U/L	46.9 U/L	Hemoglobin (Hb) concentration	11.8 g/dL	16.6 g/dL
C-reactive protein (CRP)	0.2 mg/L	13.1 mg/L	High light scatter reticulocyte (HLR) count	0.01 × 10^12^/L	0.04 × 10^12^/L
Calcium	2.2 mmol/L	2.6 mmol/L	High light scatter reticulocyte percentage	0.1%	0.9%
Cholesterol	3.6 mmol/L	8.1 mmol/L	Immature reticulocyte fraction (IRF)	0.17	0.41
Creatinine	48.7 μmol/L	105 μmol/L	Lymphocyte count	1.0 × 10^9^/L	3.4 × 10^9^/L
Cystatin C	0.67 mg/L	1.26 mg/L	Lymphocyte percentage	15.3%	44.2%
Direct bilirubin	1.0 μmol/L	4.0 μmol/L	Mean corpuscular hemoglobin (MCH)	27.7 pg	34.7 pg
Gamma glutamyltransferase (GGT)	11.5 U/L	130 U/L	Mean corpuscular hemoglobin concentration (MCHC)	32.8 g/dL	36.4 g/dL
Glucose	3.9 mmol/L	7.7 mmol/L			
Hemoglobin A1c (HbA1C)	27.8 mmol/mol	52.6 mmol/mol	Mean corpuscular volume (MCV)	82.0 fL	99.6 fL
High-density lipoprotein (HDL) cholesterol	0.8 mmol/L	2.3 mmol/L	Mean platelet volume (MPV)	7.6 fL	11.8 fL
Insulin-like growth factor 1 (IGF-1)	11.4 nmol/L	33.4 nmol/L	Mean reticulocyte volume (MRV)	89.0 fL	121 fL
Low-density lipoprotein (LDL) cholesterol	2.0 mmol/L	5.4 mmol/L	Mean sphered cell volume (MSCV)	73.2 fL	93.8 fL
Lipoprotein A (Lp(a))	4.2 nmol/L	174 nmol/L	Monocyte count	0.2 × 10^9^/L	0.9 × 10^9^/L
Phosphate	0.8 mmol/L	1.5 mmol/L	Monocyte percentage	3.2%	11.8%
Sex hormone-binding globulin (SHBG), female	19.7 nmol/L	139 nmol/L	Neutrophil count	2.1 × 10^9^/L	7.5 × 10^9^/L
SHBG, male	15.2 nmol/L	79.1 nmol/L	Neutrophil percentage	43.9%	77.0%
Testosterone, female	0.4 nmol/L	2.4 nmol/L	Platelet count	152 × 10^9^/L	381 × 10^9^/L
Testosterone, male	5.9 nmol/L	20.2 nmol/L	Platelet crit	0.15%	0.34%
Total bilirubin	4.4 μmol/L	21.3 μmol/L	Platelet distribution width (PDW)	15.6%	17.7%
Total protein	65.0 g/L	81.2 g/L	Red blood cell (RBC) count	3.8 × 10^12^/L	5.4 × 10^12^/L
Triglycerides	0.6 mmol/L	4.4 mmol/L	Red blood cell distribution width (RDW)	12.2%	15.7%
Urate	174 μmol/L	482 μmol/L	Reticulocyte count	0.02 × 10^12^/L	0.12 × 10^12^/L
Urea	3.2 mmol/L	8.4 mmol/L	Reticulocyte percentage	0.5%	2.5%
Vitamin D	15.5 nmol/L	94.1 nmol/L	White blood cell (WBC) count	4.0 × 10^9^/L	10.9 × 10^9^/L

*%ile* percentile.

The choice to use the 95% confidence interval as a measure of normalcy was made for two reasons: predefined reference ranges are often inconsistent across sources [22], and having similar numbers of out-of-range individuals allows for more direct and consistent comparisons across tests. A sensitivity analysis incorporating reference ranges is described later in the text.

**Fig. 1:**
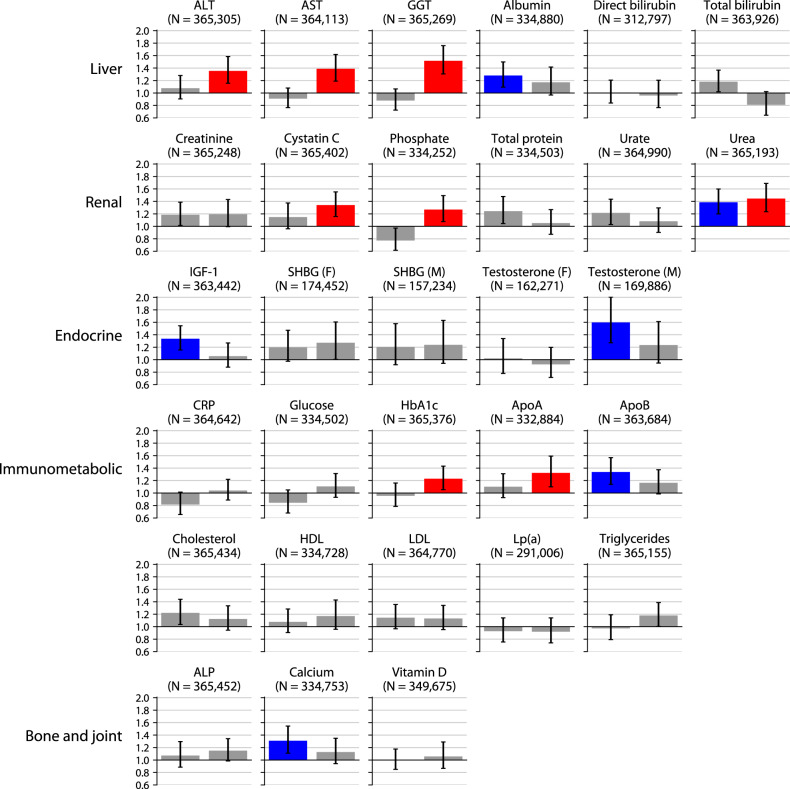
Association of blood biochemistry tests with 5-year MDD incidence. Bars indicate the adjusted odds ratio of 5-year MDD incidence among individuals in the bottom 2.5% (left) or top 2.5% (right) of each blood test, after correcting for demographic, temporal, socioeconomic, and lifestyle factors. Significant associations at 5% FDR are denoted in blue (bottom 2.5%) or red (top 2.5%). Error bars denote 95% confidence intervals. Abbreviations are defined in Table 1.

We first considered the blood biochemistry tests assayed by the UK Biobank, which encompass measures of liver, kidney, endocrine, immune, metabolic, and skeletal homeostasis. A variety of markers of abnormal liver function were associated with increased 5-year incidence of MDD (first row of Fig. 1), even after correcting for a variety of covariates including alcohol intake and body mass index (“Methods”). MDD incidence was higher among individuals with elevated (top 2.5%) levels of the liver enzymes alanine aminotransferase (ALT; adjusted odds ratio (AOR) = 1.35 [1.16, 1.58], FDR = 0.1%), aspartate aminotransferase (AST; AOR = 1.39 [1.19, 1.62], FDR = 0.05%), and gamma glutamyltransferase (GGT; AOR = 1.52 [1.31, 1.76], FDR = 0.0005%). Similar observations were made for individuals with low (bottom 2.5%) albumin levels (AOR = 1.28 [1.09, 1.50], FDR = 1%). Notably, all these associations were specific to the direction (high or low) indicative of potential liver dysfunction: low ALT, AST, and GGT and high albumin did not display any association. Two other markers of liver function, elevated direct and total bilirubin, were not associated with MDD incidence.

Among kidney function tests, high creatinine, a canonical marker of renal impairment (second row of Fig. 1), was not significantly associated with 5-year MDD incidence (AOR = 1.18 [0.98, 1.42], FDR = 20%). However, high cystatin C, considered to be a more accurate marker of renal impairment [23, 24], was associated with increased MDD incidence (AOR = 1.34 [1.16, 1.55], FDR = 0.09%), as were two other markers of renal impairment, high phosphate (AOR = 1.27 [1.08, 1.49], FDR = 2%) and high urea (also known as blood urea nitrogen; AOR = 1.39 [1.20, 1.61], FDR = 0.02%). The final two renal impairment markers tested, high urate (AOR = 1.08 [0.90, 1.30], FDR = 60%) and high total protein (AOR = 1.05 [0.87, 1.27], FDR = 70%), were not significantly associated. The lack of a statistically significant association with creatinine, urate, and total protein may reflect lack of power, as all three trend towards increased MDD incidence.

Two endocrine markers (third row of Fig. 1) were associated with MDD incidence: low insulin-like growth factor 1 (IGF-1; AOR = 1.34 [1.16, 1.55], FDR = 0.09%) and low testosterone among males (AOR = 1.60 [1.27, 2.00], FDR = 0.07%) but not among females (AOR = 1.01 [0.76, 1.33], FDR = 100%). Low IGF-1 is often used to diagnose growth hormone deficiency, since IGF-1 displays less diurnal variation than growth hormone itself [25]. The testosterone association had the largest effect size among all 57 blood tests surveyed.

In line with prior work linking depression to insulin resistance [8], we found that high levels of hemoglobin A1C (HbA1c) were associated with increased 5-year MDD incidence (AOR = 1.23 [1.05, 1.43], FDR = 4%). The only other significantly associated immunometabolic abnormalities (fourth and fifth rows of Fig. 1) were high levels of apolipoprotein A (ApoA; AOR = 1.32 [1.10, 1.59], FDR = 2%) and low levels of apolipoprotein B (ApoB; AOR = 1.34 [1.14, 1.57], FDR = 0.3%). These associations are difficult to interpret due to the absence of associations with any other lipid species (total, HDL or LDL cholesterol, Lp(a), or triglycerides). Strikingly, despite prior work linking systemic inflammation [7] with MDD, we found no link between elevations in CRP and 5-year MDD incidence (AOR = 1.06 [0.91, 1.22], FDR = 40%).

Finally, of the three markers grouped together by the UK Biobank under the “bone and joint” category (last row of Fig. 1), low calcium levels were associated with increased MDD incidence (AOR = 1.31 [1.11, 1.54], FDR = 0.9%), while alkaline phosphatase and vitamin D levels were not. This calcium association is difficult to interpret since hypocalcemia is both a feature of specific conditions like hypoparathyroidism and kidney disease and an indicator of poor health more generally: for instance, 18% of hospital inpatients and 85% of intensive care unit patients are calcium-deficient [26].

### Association of blood counts with 5-year MDD incidence

**Fig. 2:**
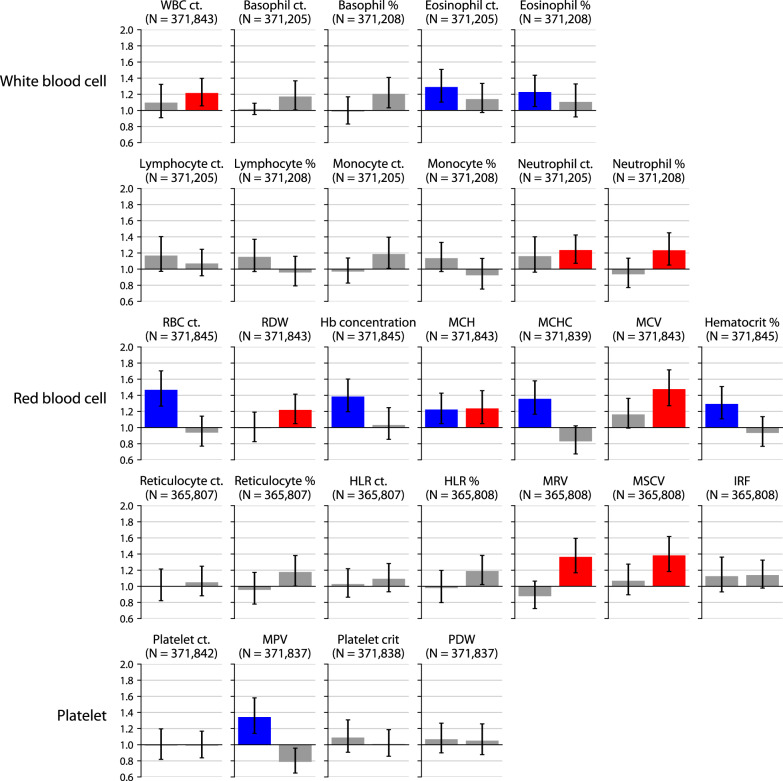
Association of blood counts with 5-year MDD incidence. Adjusted odds ratios of 5-year MDD incidence among individuals in the bottom 2.5% (left) or top 2.5% (right) of each blood test, after correcting for demographic, temporal, socioeconomic, and lifestyle factors. Significant associations at 5% FDR are denoted in blue (bottom 2.5%) or red (top 2.5%). Error bars denote 95% confidence intervals. ct. count; other abbreviations are defined in Table 1.

We next considered the association of blood cell counts with 5-year MDD incidence in the white British subcohort (Fig. 2). High white blood cell (WBC) count was associated with increased MDD incidence (AOR = 1.21 [1.06, 1.40], FDR = 3%), as was one particular WBC subset: high neutrophil count (AOR = 1.23 [1.07, 1.42], FDR = 2%) and percentage (AOR = 1.23 [1.05, 1.45], FDR = 5%) were associated, but not high basophil, eosinophil, lymphocyte, or monocyte counts or percentages.

Nine of 14 red blood cell (RBC) measures were also associated with MDD incidence. Low RBC count, an indicator of anemia, was strongly associated with incident MDD (AOR = 1.47 [1.26, 1.70], FDR = 0.002%), along with low hemoglobin concentration (AOR = 1.38 [1.19, 1.60], FDR = 0.03%) and hematocrit percentage (AOR = 1.29 [1.11, 1.51], FDR = 0.7%). MDD incidence was also associated with high mean corpuscular (RBC) volume (MCV; AOR = 1.48 [1.27, 1.71], FDR = 0.002%), an indicator of macrocytosis—an enlargement of RBCs that often co-occurs with anemia (macrocytic anemia). Thus, indicators of anemia and macrocytosis were associated with increased MDD incidence.

### Influence of the choice of percentile threshold

Given the arbitrary nature of our 2.5th percentile threshold for defining extreme test values, we next performed sensitivity analyses to determine how changing this threshold affected our results. For each of the 30 significant associations (FDR < 5%) from the main analysis, we recalculated adjusted odds ratios for 5-year MDD incidence at thresholds of 0.5, 1, 5, 10, 25, and 50% in addition to 2.5%. We cross-referenced these results against reference ranges for each test, derived from the UK Biobank, Oxford University hospital system, and published literature (“Methods”).

**Fig. 3:**
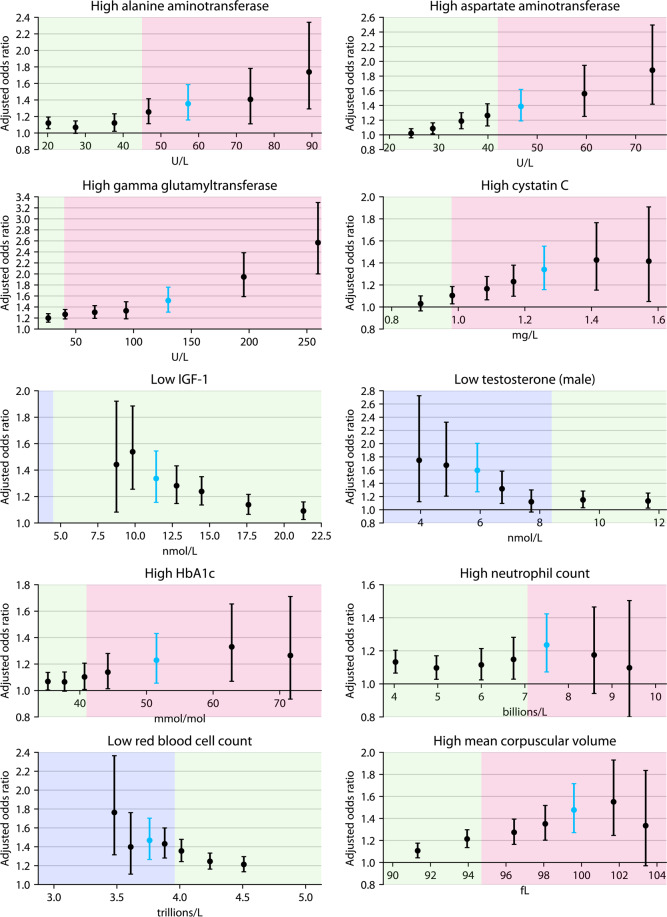
Dependence of associations on the choice of percentile threshold. Adjusted odds ratios and 95% confidence intervals for 5-year MDD incidence at seven choices of the percentile threshold (top or bottom 0.5, 1, 2.5, 5, 10, 25, and 50%) for 10 of the 30 significant associations from the main analysis. (The remaining 20 are shown in Fig. S2.) The adjusted odds ratio at the original 2.5% threshold is highlighted in light blue. Green shading denotes reference ranges (see “Methods“), with below-range values shaded blue and above-range values shaded red.

For most tests, adjusted odds ratios for MDD incidence were higher at more extreme thresholds (Fig. 3 and Fig. S2). For instance, participants with above-median (top 50%) AST had a similar incidence of MDD as those with below-median AST (AOR = 1.02 [0.96, 1.08], FDR = 50%), but those with the highest 0.5% of AST were at a substantially greater risk of MDD than the bottom 99.5% (AOR = 1.88 [1.42, 2.50], FDR = 0.009%). Even for tests that did not follow this pattern, associations were relatively robust to the choice of threshold, including thresholds both inside and outside the tests’ reference ranges (with low IGF-1 being a notable exception: very few individuals in the cohort met the Oxford criteria for being out of range).

### Trans-ancestry replication

We tested the 30 significant associations from the white British subcohort for replication in participants of other ancestries (i.e. non-British white, mixed, Asian or Asian British, Black or Black British, Chinese, or other ethnic group; *N* = 50,759). We noted much lower power than in the main cohort, with only low RBC count reaching statistical significance (AOR = 2.10 [1.48, 2.99], FDR = 0.5%). Nonetheless, 20 of the 30 adjusted odds ratios from the replication analysis were greater than 1 (with the other 10 being slightly less than 1 and with large confidence intervals). These 20 include almost all of the key associations discussed in the preceding sections, including high AST and GGT, low albumin, high cystatin C, high phosphate, high urea, low IGF-1 and testosterone, high HbA1C, high white blood cell count, high neutrophil count and percentage, low red blood cell count, and high MCV. As a group, the 30 adjusted odds ratios were significantly greater than 1 (Wilcoxon *p* = 0.0005), with a median odds ratio of 1.235 (Table S3).

## Discussion

In this study, we investigated whether abnormal results (<2.5th or >97.5th percentile) on each of 57 blood tests were prospectively associated with 5-year MDD incidence in the community-based UK Biobank cohort. We found consistent associations across ancestries and percentile thresholds with markers of specific types of somatic dysfunction: liver and renal abnormalities, growth hormone and testosterone deficiency, insulin resistance (even with adjustment for body mass index), anemia, and macrocytosis. While our study is far from the first to explore blood markers of MDD incidence, our use of the UK Biobank, a large cohort with detailed sociodemographic and lifestyle information, enables methodological consistency across markers and extensive covariate correction and sensitivity analyses.

Six aspects of our results are particularly noteworthy. First, all 30 significant associations were in the direction of increased, rather than decreased, MDD incidence. In other words, abnormalities in the blood tests we surveyed were always deleterious or neutral from the perspective of MDD risk, never protective.

Second, many markers of liver and renal dysfunction were associated with increased MDD incidence. While both chronic liver [27, 28] and chronic kidney [29, 30] disease have been cross-sectionally associated with MDD, we believe this study is the first to show a prospective association between these markers and MDD. Notably, bilirubin was not associated with MDD, perhaps because it is a less specific indicator of liver dysfunction than the transaminases ALT and AST, often reflecting excessive heme breakdown, Gilbert’s syndrome (a benign hyperbilirubinemia present in 5% of the population), or bile duct obstruction [31] instead.

Third, we find links between MDD incidence and multiple types of endocrine dysregulation. The association with low IGF-1 is consistent with prior work suggesting that many people with adult-onset growth hormone deficiency exhibit an MDD phenotype that is responsive to growth hormone therapy [32] though the observation that MDD patients often have *high* IGF-1 levels [33–36] suggests that IGF-1 dysregulation in either direction may be harmful. IGF-1 deficiency is intimately linked to insulin resistance [37], and the associations we observe with low IGF-1 and elevated HbA1C are consistent with a key role for metabolic dysregulation in MDD, or at least certain subtypes thereof [8, 38]. Meanwhile, the testosterone association is consistent with the increased prevalence of diagnosed MDD and dysthymia among testosterone-deficient middle aged and older men [39, 40], and with the antidepressant effects of testosterone replacement therapy observed in randomized clinical trials [41, 42].

Fourth, numerous red blood cell properties were associated with increased MDD incidence, including several indicative of anemia and macrocytosis. While prior cross-sectional studies have associated anemia with depression [43, 44], this study is, to our knowledge, the first to show a prospective association between markers of anemia and MDD. The association with macrocytosis is difficult to interpret, as it often results from vitamin B_12_ deficiency, folate deficiency, hypothyroidism, or liver disease [45], and some of these processes, rather than macrocytosis itself, could be responsible for the association.

Fifth, we find no significant association between MDD incidence and elevated CRP, in contrast to some prior studies. The discordance between CRP and neutrophil count, which we do find associated with MDD, is consistent with the distinct biology of these two markers [46] and the poor correlation [47–49] between CRP and neutrophil-to-lymphocyte ratio, another common inflammatory measure. It could also reflect CRP’s correlation with body mass index [50, 51], a covariate in our model.

Sixth, adjusted odds ratios tend to become larger as the percentile threshold becomes more extreme. In other words, the less healthy one is—from the perspective of clinical laboratory tests—the higher one’s risk of developing MDD. This is consistent with the notion that many non-psychiatric comorbidities are key risk factors for MDD across the lifespan [52]. However, many of our hits are associated with MDD even at thresholds well inside the reference range, suggesting that even “sub-clinical” abnormalities in laboratory measures, common among individuals without overt comorbidities, are still clinically meaningful from the perspective of MDD risk. One process which may underlie such sub-clinical abnormalities, particularly in our mid-to-late-life cohort, is cellular senescence, which has known etiological links to MDD [53, 54].

Despite its methodological strengths, this work has several limitations. First, despite our extensive covariate correction, residual confounding may still influence some of our associations. Second, our 57 blood markers represent only a small fraction of those ever tested for association with MDD, and likely are not the best markers possible: for instance, peripheral biomarkers of neuroinflammation [55] may be more strongly associated with MDD than CRP [56]. On the other hand, the fact that most are already widely used in the clinic does have the advantage of making our findings more immediately translational (though of course, just because individual laboratory measures are associated with MDD does not automatically make them clinically useful as screening tools). Third, there are likely unaccounted for covariates that were not or could not be included, and those that were included may not be fully reliable, particularly self-reported measures of substance use.

Overall, we find that markers of liver and kidney dysfunction, growth hormone and testosterone deficiency, innate immunity, insulin resistance, anemia, and macrocytosis are associated with 5-year MDD incidence in a large community-based cohort, supporting roles for diverse somatic processes in MDD onset. Our results suggest that interventions to improve liver and kidney function, raise growth hormone and testosterone levels, reduce systemic inflammation, improve glycemic control and treat anemia may represent viable strategies for preventing certain cases of new-onset MDD.

## Supplementary information

Supplement
