# Supplementary material for: Clinical laboratory tests and five-year incidence of major depressive disorder: a prospective cohort study of 433,890 participants from the UK Biobank
Source: Transl Psychiatry. 2021 Jul 7;11:380. doi: 10.1038/s41398-021-01505-5 (PMC8263616; doi:10.1038/s41398-021-01505-5)
Supplement: Supplementary file 1 — Supplement [file 41398_2021_1505_MOESM1_ESM.pdf]

Supplement

Characteristic	Value	% of controls (N = 428,356)	% of cases (N = 5534)
**Age**	35 to 44	10.1%	11.3%
	45 to 54	27.9%	31.3%
	55 to 64	42.3%	39.5%
	65 to 74	19.6%	17.8%
**Sex**	Female	53.2%	61.5%
	Male	46.8%	38.5%
**Ancestry**	White British	88.3%	87.7%
	Other white	5.9%	6.5%
	Mixed	0.6%	0.8%
	Asian or Asian British	2.0%	1.8%
	Black or Black British	1.6%	1.5%
	Chinese	0.3%	0.1%
	Other ethnic group	0.9%	1.1%
**Educational qualifications**	College or University degree	32.6%	24.6%
	A levels/AS levels or equivalent	11.1%	9.8%
	O levels/GCSEs or equivalent	21.2%	21.1%
	CSEs or equivalent	5.3%	7.2%
	NVQ or HND or HNC or equivalent	6.6%	7.0%
	Other professional qualifications	5.2%	5.1%
**Pre-tax household income**	<£18k	18.3%	29.4%
	£18-31k	21.8%	20.5%
	£31-52k	22.6%	19.2%
	£52-100k	17.9%	12.5%
	>£100k	4.8%	1.9%
**Employment status**	Paid employment or self-employed	58.1%	51.3%
	Retired	33.7%	31.2%
	Looking after home and/or family	2.7%	3.5%
	Unable to work	2.3%	9.9%
	Unemployed	1.5%	2.1%
	Doing unpaid or voluntary work	0.4%	0.4%
	Full or part-time student	0.3%	0.3%
**Townsend Deprivation Index**	Lowest quintile (least deprived)	20.1%	14.7%
	Second-lowest quintile	20.1%	16.0%
	Middle quintile	20.0%	18.3%
	Second-highest quintile	20.0%	21.0%
	Highest quintile (most deprived)	19.9%	30.0%
**Index of Multiple Deprivation**	Lowest quintile (least deprived)	20.1%	13.6%
	Second-lowest quintile	20.0%	16.8%
	Middle quintile	20.0%	17.7%
	Second-highest quintile	20.0%	21.4%
	Highest quintile (most deprived)	19.8%	30.4%
**Alcohol**	Daily or almost daily	20.5%	16.5%
	Three or four times a week	23.6%	17.7%
	Once or twice a week	26.1%	24.9%
	One to three times a month	11.0%	13.0%
	Special occasions only	11.1%	14.8%
**Smoking**	Never	55.2%	46.1%
	Previous	34.5%	34.3%
	Current	9.8%	18.8%
**Body mass index**	Underweight (<18)	0.3%	0.5%
	Normal (18-25)	33.2%	26.9%
	Overweight (25-30)	42.9%	39.6%
	Obese (over 30)	23.7%	33.1%

Table S1: Covariate distributions among incident MDD cases and controls.

Blood biochemistry	Bottom	Top
Alanine aminotransferase (ALT)	10 U/L	45 U/L
Albumin	32 g/L	50 g/L
Alkaline phosphatase (ALP)	30 U/L	130 U/L
Aspartate aminotransferase (AST)	15 U/L	42 U/L
C-reactive protein (CRP)	0 mg/L	5 mg/L
Calcium	2.2 mmol/L	2.6 mmol/L
Creatinine	49 µmol/L	90 µmol/L
Cystatin C	0.51 mg/L	0.98 mg/L
Direct bilirubin	0 µmol/L	5 µmol/L
Gamma glutamyltransferase (GGT)	15 U/L	40 U/L
Hemoglobin A1c (HbA1C)	20 mmol/mol	41 mmol/mol
Insulin growth factor-like 1 (IGF-1)	4.5 nmol/L	31.7 nmol/L
Phosphate	0.7 mmol/L	1.45 mmol/L
Sex hormone-binding globulin (SHBG), female	16 nmol/L	148 nmol/L
SHBG, male	14 nmol/L	78 nmol/L
Testosterone, female	0.5 nmol/L	2.6 nmol/L
Testosterone, male	8.4 nmol/L	28.7 nmol/L
Total bilirubin	0 µmol/L	21 µmol/L
Total protein	60 g/L	80 g/L
Urate	150 µmol/L	420 µmol/L
Urea	2.5 mmol/L	9.2 mmol/L
Vitamin D	50 nmol/L	N/A

Blood count	Bottom	Top
Basophil count	0.01×10^9/L	0.13×10^9/L
Basophil percentage	0.17%	1.37%
Eosinophil count	0.03×10^9/L	0.77×10^9/L
Eosinophil percentage	0.75%	8.06%
Hematocrit percentage	35.39%	47.19%
Hemoglobin (Hb) concentration	12.14 g/dL	16.27 g/dL
Immature reticulocyte fraction (IRF)	0.163	0.362
Lymphocyte count	0.65×10^9/L	4.25×10^9/L
Lymphocyte percentage	18.39%	44.42%
Mean corpuscular hemoglobin (MCH)	25.69 pg	32.95 pg
Mean corpuscular hemoglobin concentration (MCHC)	33.34 g/dL	35.47 g/dL
Mean corpuscular volume (MCV)	76.94 fL	94.7 fL
Mean platelet volume (MPV)	7.54 fL	11.24 fL
Mean reticulocyte volume (MRV)	102.73 fL	124.89 fL
Monocyte count	0.17×10^9/L	1.21×10^9/L
Monocyte percentage	4.69%	12.66%
Neutrophil count	1.47×10^9/L	7.06×10^9/L
Neutrophil percentage	41.71%	73.74%
Platelet count	169×10^9/L	397×10^9/L
Red blood cell (RBC) count	3.96×10^{12}/L	5.50×10^{12}/L
Red blood cell distribution width (RDW)	12.1%	15.2%
Reticulocyte count	0.02×10^{12}/L	0.11×10^{12}/L
Reticulocyte percentage	0.45%	2.28%
White blood cell (WBC) count	3.53×10^9/L	9.57×10^9/L

Table S2: Reference ranges used in our analyses.

Significant association among white British	AOR, white British	AOR, other ancestries
High alanine aminotransferase (ALT)	1.35 [1.16, 1.58]	0.98 [0.60, 1.61]
Low albumin	1.28 [1.09, 1.50]	1.37 [0.90, 2.07]
High apolipoprotein A (ApoA)	1.32 [1.10, 1.59]	1.22 [0.74, 2.00]
Low apolipoprotein B (ApoB)	1.34 [1.14, 1.57]	1.06 [0.66, 1.71]
High aspartate aminotransferase (AST)	1.39 [1.19, 1.62]	1.17 [0.75, 1.83]
Low calcium	1.31 [1.11, 1.54]	0.93 [0.55, 1.57]
High cystatin C	1.34 [1.16, 1.55]	1.41 [0.97, 2.06]
High gamma glutamyltransferase (GGT)	1.52 [1.31, 1.76]	1.28 [0.84, 1.95]
High hemoglobin A1c (HbA1C)	1.23 [1.05, 1.43]	1.21 [0.80, 1.83]
Low insulin growth factor-like 1 (IGF-1)	1.34 [1.16, 1.55]	1.48 [1.02, 2.15]
High phosphate	1.27 [1.08, 1.49]	1.45 [0.95, 2.21]
Low testosterone (male)	1.60 [1.27, 2.00]	1.33 [0.69, 2.57]
High urea	1.44 [1.23, 1.69]	1.27 [0.83, 1.95]
Low urea	1.39 [1.20, 1.60]	0.86 [0.52, 1.43]
Low eosinophil count	1.29 [1.10, 1.51]	0.77 [0.44, 1.35]
Low eosinophil percentage	1.23 [1.05, 1.44]	0.84 [0.49, 1.44]
Low hematocrit	1.29 [1.11, 1.51]	1.81 [1.23, 2.66]
Low hemoglobin concentration	1.38 [1.19, 1.60]	1.44 [0.94, 2.19]
High mean corpuscular hemoglobin (MCH)	1.24 [1.05, 1.46]	1.10 [0.71, 1.70]
Low mean corpuscular hemoglobin (MCH)	1.22 [1.05, 1.43]	0.90 [0.53, 1.55]
Low mean corpuscular hemoglobin concentration (MCHC)	1.36 [1.17, 1.58]	0.80 [0.46, 1.41]
High mean corpuscular volume (MCV)	1.48 [1.27, 1.71]	1.19 [0.77, 1.81]
Low mean platelet volume (MPV)	1.34 [1.14, 1.58]	1.45 [0.95, 2.22]
High mean reticulocyte volume (MRV)	1.36 [1.17, 1.59]	0.90 [0.54, 1.50]
High mean sphered cell volume (MSCV)	1.38 [1.18, 1.62]	0.93 [0.57, 1.54]
High neutrophil count	1.23 [1.07, 1.42]	1.38 [0.96, 1.99]
High neutrophil percentage	1.23 [1.05, 1.45]	1.42 [0.94, 2.14]
Low red blood cell count	1.47 [1.26, 1.70]	2.10 [1.48, 2.99]
Low red blood cell distribution width (RDW)	1.22 [1.05, 1.41]	1.25 [0.76, 2.03]
High white blood cell count	1.21 [1.06, 1.40]	1.26 [0.87, 1.82]

Table S3: Adjusted odds ratios (AORs) in the main cohort (white British) and replication cohort (other ancestries) for the 30 significant associations from the main analysis.

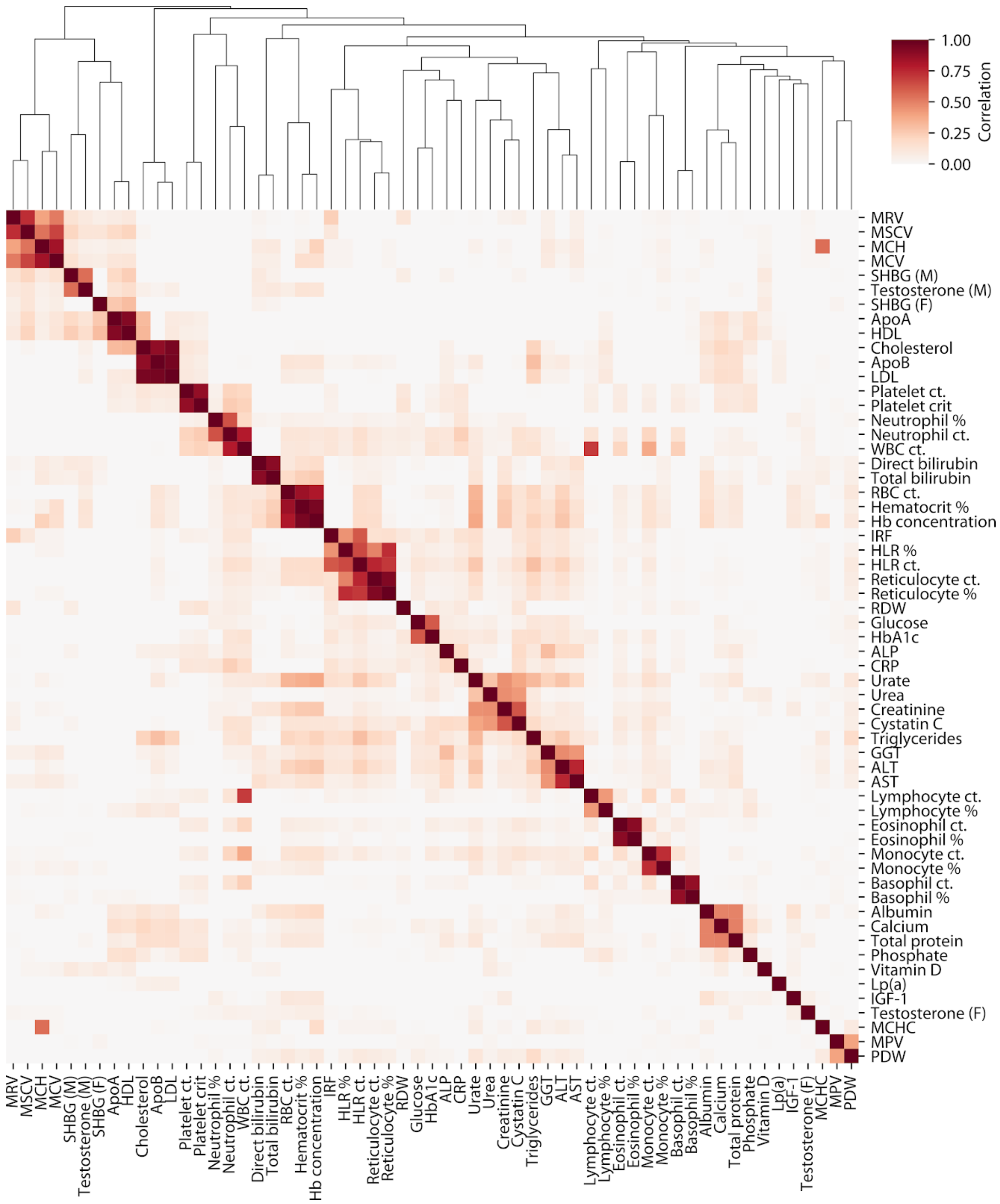

Figure S1: Pearson correlation coefficients for each pair of blood tests. For each pair of tests, the correlation is taken across all participants in the main white British cohort with non-missing data for both tests.

Figure S2 (next two pages): Continuation of Figure 3. Three tests, apolipoproteins A and B and mean sphered cell volume, have white backgrounds because they had no reference ranges listed in either the UK Biobank or the Oxford University hospital system.

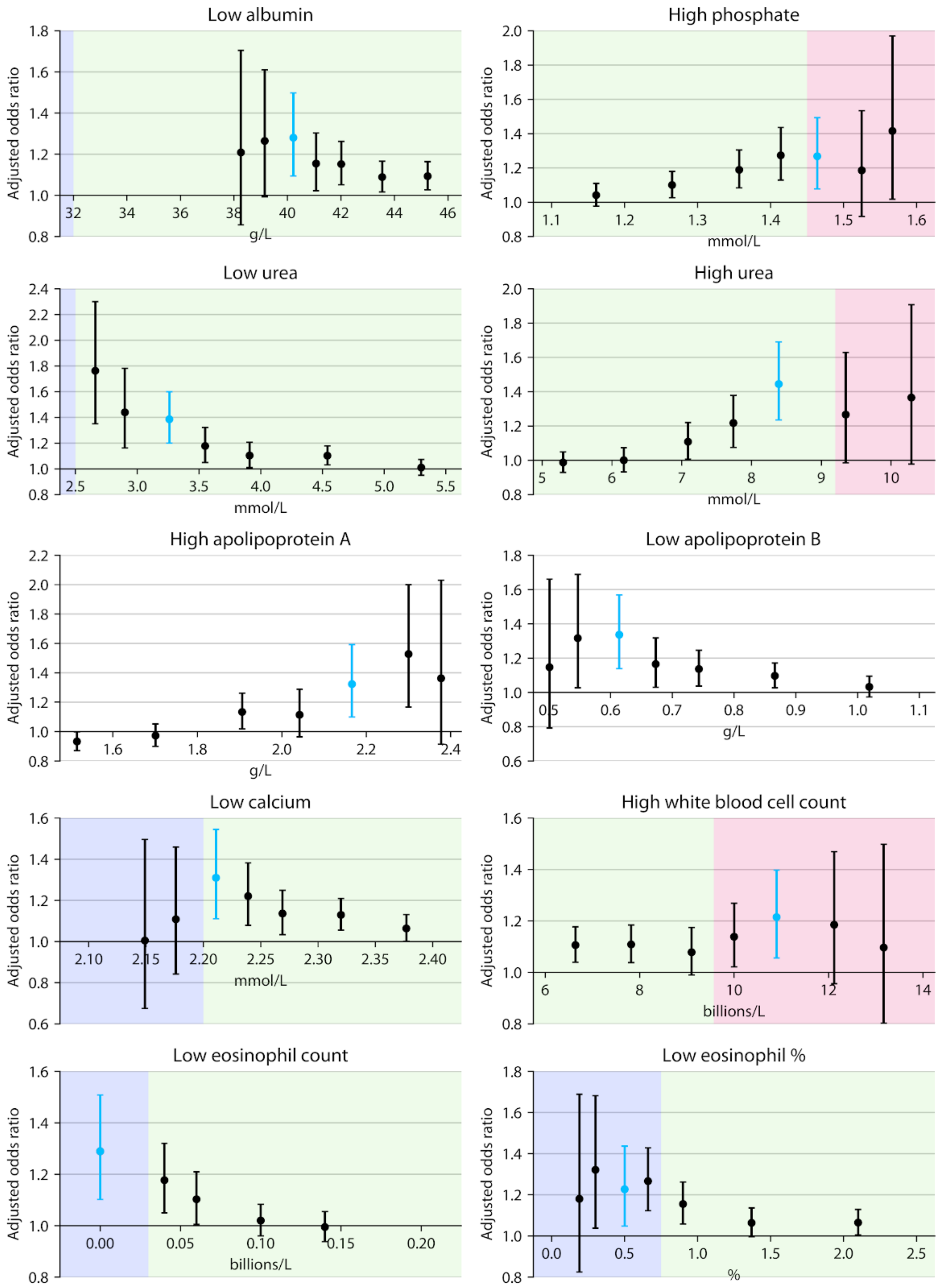
Low albumin
Adjusted odds ratio
g/L
High phosphate
Adjusted odds ratio
mmol/L
Low urea
Adjusted odds ratio
mmol/L
High urea
Adjusted odds ratio
mmol/L
High apolipoprotein A
Adjusted odds ratio
g/L
Low apolipoprotein B
Adjusted odds ratio
g/L
Low calcium
Adjusted odds ratio
mmol/L
High white blood cell count
Adjusted odds ratio
billions/L
Low eosinophil count
Adjusted odds ratio
billions/L
Low eosinophil %
Adjusted odds ratio
%

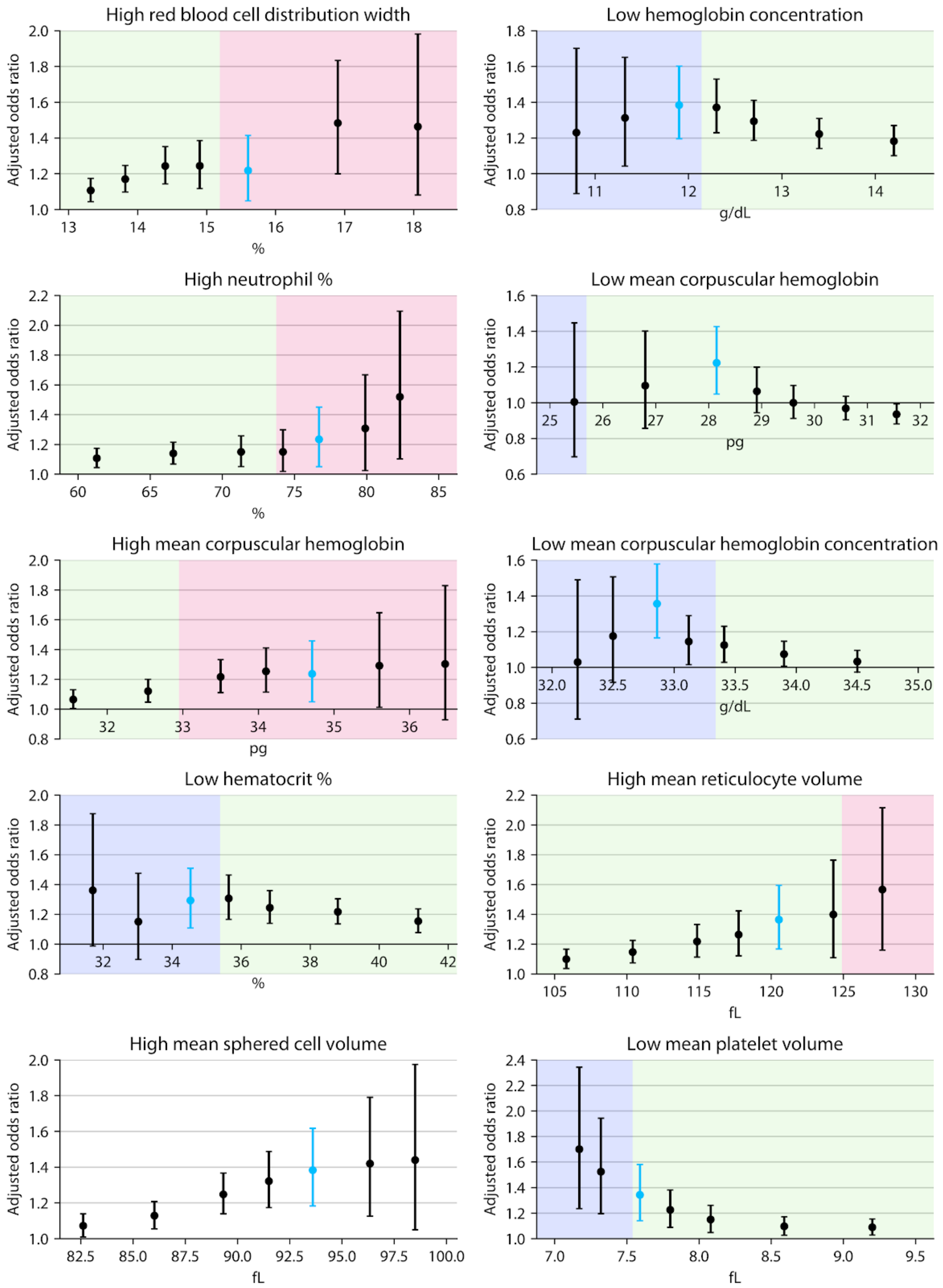
High red blood cell distribution width
Adjusted odds ratio
%
Low hemoglobin concentration
g/dL
High neutrophil %
%
Low mean corpuscular hemoglobin
pg
High mean corpuscular hemoglobin
pg
Low mean corpuscular hemoglobin concentration
g/dL
Low hematocrit %
%
High mean reticulocyte volume
fL
High mean sphered cell volume
fL
Low mean platelet volume
fL
